# Success rates of intensive aphasia therapy: real-world data from 448 patients between 2003 and 2020

**DOI:** 10.1007/s00415-024-12429-7

**Published:** 2024-05-20

**Authors:** Dorothea Peitz, Beate Schumann-Werner, Katja Hussmann, João Pinho, Hong Chen, Ferdinand Binkofski, Walter Huber, Klaus Willmes, Stefan Heim, Jörg B. Schulz, Bruno Fimm, Cornelius J. Werner

**Affiliations:** 1 Department of Neurology, Medical Faculty, RWTH Aachen University, Pauwelsstrasse 30, 52074 Aachen, Germany; 2 Institute of Cognitive Neurology and Dementia Research, Otto-Von-Guericke-University, Magdeburg, Germany; 3 Section Clinical Cognitive Sciences, Department of Neurology, Medical Faculty, RWTH Aachen University, Aachen, Germany; 4 Department of Psychiatry, Psychotherapy and Psychosomatics, Medical Faculty, RWTH Aachen University, Aachen, Germany; 5 Institute of Neuroscience and Medicine (INM-1), Research Centre Jülich, Jülich, Germany; 6 JARA-BRAIN Institute Molecular Neuroscience and Neuroimaging, Research Centre Jülich GmbH, Jülich, Germany; 7 JARA-BRAIN Institute Molecular Neuroscience and Neuroimaging, RWTH Aachen University, Aachen, Germany; 8 Department of Neurology and Geriatrics, Johanniter Hospital Stendal, Hansestadt Stendal, Germany

**Keywords:** Aphasia, Treatment outcome, Impairment-based therapy, Responder rates

## Abstract

**Background:**

Aphasia is a devastating consequence after stroke, affecting millions of patients each year. Studies have shown that intensive speech and language therapy (SLT) is effective in the chronic phase of aphasia. Leveraging a large single-center cohort of persons with aphasia (PWA) including patients also in the subacute phase, we assessed treatment effects of intensive aphasia therapy in a real-world setting.

**Methods:**

Data were collected at the Aachen aphasia ward in Germany between 2003 and 2020. Immediate treatment responses across different language domains were assessed with the Aachen Aphasia Test (AAT) using single-case psychometrics, conducted before and after 6–7 weeks of intensive SLT (10 h per week, median (IQR) dosage = 68 (61–76)). We adjusted for spontaneous recovery in subacute patients. Differential treatment effects between subgroups of chronicity and predictors of therapy response were investigated.

**Results:**

A total of 448 PWA were included (29% female, median (IQR) age = 54 (46–62) years, median (IQR) time post-onset = 11 (6–20) months) with 12% in the early subacute, 15% in the late subacute and 74% in the chronic phase of aphasia. The immediate responder rate was 59%. Significant improvements in all AAT subtests und subscales were observed hinting at broad effectiveness across language domains. The degree of therapy-induced improvement did not differ between the chronicity groups. Time post-onset, dosage of therapy and aphasia severity at the beginning of treatment were predictors of immediate treatment response.

**Discussion:**

Intensive therapy protocols for aphasia after stroke are yielding substantial responder rates in a routine clinical setting including a wide range of patients.

**Supplementary Information:**

The online version contains supplementary material available at 10.1007/s00415-024-12429-7.

## Introduction

Aphasia is an acquired language disorder commonly occurring after ischemic or hemorrhagic stroke in the dominant hemisphere. It affects about 40% of stroke survivors [1] and persists to 1 year or more after stroke in up to 30% [2]. Aphasia severely influences daily activities and quality of life [3] and decreases the likelihood to return to work [4]. The resulting high economic burden of poststroke aphasia [5] calls for optimal healthcare services for persons with aphasia (PWA). However, the development and implementation of intensive speech and language therapy (SLT) are cost-intensive [6]. Limited resources and higher prevalence of chronic stroke lead to an ongoing debate about the effectiveness and optimal use of aphasia therapy between different stakeholders.

Multiple studies have shown the effectiveness of intensive SLT for the subacute [7–10] and chronic [11, 12] phase of aphasia. Although evidence supports the efficacy of SLT with high dose and intensity [13–15], a large discrepancy between research studies and routine clinical services remains [16–18]. One example for delivering high dose treatment in a real-world setting is the Intensive Comprehensive Aphasia Program (ICAP). A recent review summarized that improvements in naming were the most robust findings of therapy outcomes in ICAP research [19]. However, some studies indicate that other language domains apart from speech production (i.e., writing, reading, and comprehension), functional communication and quality of life can also improve in PWA with chronic aphasia [20]. More research is needed in intensive SLT from real-world settings which leverages large datasets and also includes PWA in the subacute phase of aphasia.

In this observational study with a single-group pre-posttest design, we investigated the outcome of personalized, impairment-based, intensive SLT similar to an ICAP, conducted under real-world clinical conditions in a large sample of PWA in the subacute and chronic phase. Our primary objective was the examination of immediate treatment-induced improvements, evaluated with a validated and standardized outcome measure (Aachen Aphasia Test, AAT [21]) which allows comparisons at the group level but also psychometric single-case analysis [22]. Data from a historical control group [23] enabled the comparison with a group of untreated PWA in the subacute phase. We could also individually correct therapy gains for expected spontaneous recovery for PWA in the subacute phase in order to draw conclusions about the genuine effects of SLT above and beyond spontaneous recovery. This correction allowed us to estimate responder rates based on performance data of individual PWA. As secondary objectives, we looked for differential treatment effects between the subgroups of chronicity and the relationship between routinely available demographic and behavioral variables and therapy outcome.

## Materials and methods

### Participants and procedure

The database of this study contains information on all inpatients who were treated at a 12-bed aphasia ward at the University Hospital in Aachen, Germany, between 2003 and 2020. The PWA came from all parts of Germany in order to complete an intensive SLT lasting 6 to 7 weeks per treatment cycle. The aphasia treatment program was established at Aachen University Hospital in 1985 and has been described extensively elsewhere [24]. At the beginning of the treatment cycle, the main therapist determines the therapy targets for each patient individually based on initial linguistic assessments and evidence-based approaches. The treatment comprises at least 10 h of SLT per week including one-on-one (7 h/week) and group sessions (3 h/week). The overall number of all SLT sessions of the treatment cycle (dosage SLT) is available for each patient in our database (median (IQR) = 68 h (61–76)). Depending on the PWA’s symptoms, the treatment protocol is supplemented with neuropsychological training, physiotherapy, music therapy and/or other creative or communicative group sessions (Table 1). In addition, counselling of relatives and caregivers is offered on a regular basis.

**Table 1:** Therapy data of the entire cohort and results of univariate analyses for responders vs. nonresponders

	Entire cohort (*n* = 448)	Responders^a^ (*n* = 265)	Nonresponders^a^ (*n* = 183)	*p* value	Effect size (95% CI)
Duration of treatment cycle (days)	46 (46–47)	46 (46–47)	46 (46–47)	0.732	–
Speech and language therapy in hours					
One-on-one sessions with primary therapist	33 (31–34)	33 (32–35)	33 (31–34)	0.064	–
Group sessions	18 (17–19)	18 (17–19)	18 (17–19)	0.208	–
Computer-based SLT	7 (0–12)	8 (1–12)	6 (0–11)	0.004*	0.16 (0.05–0.26)
SLT in research projects or delivered by supervised students	10 (5–15)	10 (5–15)	8 (4–14)	0.067	–
Dosage of SLT	68 (61–76)	69 (62–78)	65 (59–74)	0.001*	0.18 (0.07–0.28)
Other therapies in hours					
Neuropsychological training	1 (0–4)	1 (0–3)	1 (0–4)	0.928	–
Physiotherapy	32 (25–39)	32 (25–39)	33 (25–39)	0.981	–
Music therapy	5 (2–8)	5 (2–8)	5 (3–8)	0.545	–
Other creative or communicative group sessions	8 (5–11)	8 (5–12)	8 (5–11)	0.809	–

Values are shown as median (interquartile range). Univariate analyses with Mann–Whitney *U* test, two-tailed. Effect sizes with 95% CI, only reported for comparisons with *p* ≤ 0.05 (Glass rank biserial correlation coefficient *r*_g_)

*CI* confidence interval, *SLT* Speech and language therapy

^a^Responders and nonresponders after individual correction for spontaneous recovery with the mean change scores of the historical control group

^*^p < 0.05

The database was filtered according to the following inclusion criteria: (i) stroke with vascular etiology, i.e., ischemia, hemorrhage or combination of both, (ii) symptoms of aphasia with or without apraxia of speech as the primary focus of treatment, (iii) complete standardized language assessment with the AAT [21] at the beginning and at the end of the treatment, (iv) diagnosis of aphasia at pre-treatment AAT, (v) start of treatment in the subacute or chronic phase of aphasia, i.e., more than 6 weeks post-onset, (vi) first treatment cycle of each patient meeting all previous conditions, and (vii) no stroke recurrence or other serious illnesses during the treatment cycle. The remaining PWA was classified according to the treatment cycle time point after stroke into the following subgroups: (i) early subacute group (between 6 weeks and 3 months), (ii) late subacute group (between 4 and 6 months) and (iii) chronic group (7 months or more). This classification of subgroups follows the time points of assessment of a prior study on the course of spontaneous recovery [23].

We collected demographics (age at onset, sex, handedness, education), stroke characteristics (time post-onset, stroke subtype, localization of stroke), aphasia characteristics (aphasia syndrome, aphasia severity) and performance in activities of daily living (Barthel Index) at the beginning of the treatment cycle from medical records and initial assessment sessions.

### Standard protocol approvals, registrations, and patient consents

The study was approved by the local Ethics Committee of the Medical Faculty of RWTH Aachen University (vote number: EK 038/20). No informed consent was required.

### Outcome measures and correction for spontaneous recovery

In Germany, the AAT is the gold standard for the diagnosis and classification of aphasia, and determination of severity. Crucially, it allows for valid psychometric single-case analysis of improvement [22]. We used the AAT to evaluate treatment-induced improvement between pre- and post-treatment assessment in all language modalities (five subtests) and the spontaneous speech profile (six rating subscales, see Supplementary methods) [25]. The treating therapist administered the AAT at the beginning of the treatment. In order to reduce bias towards improvement, a different person assessed the patient after treatment, usually the day before discharge. For exploratory analyses, we additionally collected data from a follow-up AAT for a subgroup of PWA. These data were not sampled systematically and were available to us only if PWA came back for a second treatment cycle.

For each AAT subtest, we calculated the difference in raw scores from pre- to post-treatment and from post-treatment to follow-up assessment (change scores). Raw scores were preferred over standard scores to avoid an over- or underestimation at the extremes of the scales due to skewed distributions in the normative sample for the AAT [21]. We additionally employed standardized *T* scores in order to calculate the AAT profile level (i.e., reliability weighted average of all subtest *T* scores) as an overall quantitative estimate of the severity of language impairment, and the change in profile level. The pre-treatment profile level was used to determine aphasia severity at the beginning of therapy [22].

Due to the data source coming from a real-world clinical workflow at the Aachen aphasia ward, a matched control group of PWA without or with non-intense therapy does not exist for this study. However, previous studies indicate the superiority of intensive SLT for PWA in the chronic phase of aphasia (> 6 months post-onset) over low-frequency regimes [11, 12, 14]. This superiority cannot be assumed for the PWA in the subacute phase of aphasia of this study, however, because of possible spontaneous recovery. In order to take this into account, we used the data from a historical sample of PWA with no access to systematically conducted SLT during the first 7 months after stroke [23]. Leveraging this historical data, we could control for possible improvements without therapy. We did this in two ways, one group-oriented and one individual PWA-oriented approach.

In the group-oriented approach, we compared standardized mean differences (unbiased Cohen’s *d*) in change scores of the AAT subtests and profile level between this study and the historical control group. This analysis was conducted separately for the early subacute and the late subacute phase. Since there are considerable differences in the samples (i.e., size and distribution) and the time points of AAT assessments between this study and the historical control study, we made a few adjustments to the calculations in order to make the samples as comparable as possible (see ‘Statistical Analysis’).

In the individual PWA-oriented approach, we used the differences of the subtests’ raw scores and the profile level of each PWA in this study and corrected them for expected spontaneous recovery, as published previously [26] (see Supplementary methods, Supplementary Table 1 and 2). Briefly, we subtracted the average raw score and profile level differences achieved by PWA with the same aphasia syndrome and time post-onset in the historical sample on spontaneous recovery [23] from the individual differences observed in our study. In order to achieve corrected post-treatment scores for the statistical analyses on the group level and for the secondary analyses, we added the adjusted score differences to the pre-treatment scores. These adjustments were possible only for the AAT subtests and the profile level because of the ordinal scale of the 6-point spontaneous speech subscales (0–5). The correction for spontaneous recovery was also not performed for the comparisons between post-treatment and follow-up AAT. Using only mean change scores from the historical control group for the individual correction might not capture all the interindividual variability of a cohort with PWA. The variability in the different courses of language gains in the subacute phase can be seen in the data of the historical control group (see Supplementary Table 1). Correlation coefficients between the AAT assessments at 1 and 4 months post-onset and the assessments at 4 and 7 months post-onset in the historical control group support the assumption that the variability is higher in the early subacute phase than in the late subacute phase. This is evident in the larger discrepancy between the intraclass correlation coefficients for absolute agreement (ICC(A,1)) and the intraclass correlation coefficients for consistency (ICC(C,1)) between 1 and 4 months post-onset than between 4 and 7 months post-onset. However, all correlation coefficients are still high, which speaks against arbitrary courses of recovery (see Supplementary Table 3). Still, in order to avoid an over- or underestimation of the spontaneous recovery in this study, we additionally calculated responder rates in our cohort using (i) the first quartile (Q1) and (ii) the third quartile (Q3) of the raw score and profile level change scores from the historical control group instead of the mean change scores. In this way, we also obtained ‘liberal’ (using Q1 for correction) and ‘conservative’ (using Q3 for correction) responder rates after individual correction for spontaneous recovery (see Supplementary methods).

We finally compared the score differences (with and without correction) with the corresponding one-tailed critical differences for significant improvement (see Supplementary Table 4) for each subtest and the profile level according to standard procedures for the AAT [8]. Two-tailed critical differences were used for the investigation of individual changes between post-treatment and follow-up AAT. For the spontaneous speech subscales, a difference of two rating points was considered as significant change, well beyond what could be expected as a random change based on interrater agreement studies for the AAT [8]. If PWA showed significant improvement from pre- to post-treatment AAT in at least one of the subtests or the profile level or one of the spontaneous speech subscales, they were classified as responders. The percentage of responders to intensive therapy was the main outcome parameter.

In an additional exploratory analysis, we examined the long-term stability of language gains in a subgroup of patients with an available follow-up AAT. For this analysis, we compared the post-treatment AAT at discharge with the follow-up AAT on the individual level only without any correction for spontaneous recovery of the score differences.

### Missing data and outliers

If an independent variable was missing for more than 10% of PWA, we assessed the importance of this variable based on (i) the correlation between the independent variable and the outcome measure for all complete cases in this study and (ii) existing literature and clinical experience. If our data showed no correlation and we found no strong evidence for a relationship in the literature, we excluded this variable from further statistical analyses. In our dataset, we had more than 10% missing data on handedness and education. Correlation analyses did not indicate any significant relationships with therapy outcome (see Supplementary Table 5), in accordance with previous literature [11, 27, 28]. Consequently, handedness and education were removed from further statistical analyses.

In addition, we removed extreme outliers which were defined as three times the interquartile range (IQR) below the first quartile or above the third quartile. According to this definition, time post-onset contained *n* = 10 extreme outliers with more than 62.5 months post-onset, which were excluded from further analyses.

### Statistical analysis

Median values and interquartile ranges are reported for all continuous variables because the data were not normally distributed, tested with Shapiro–Wilk tests (*p* < 0.05). We report numbers and proportions for categorical variables.

Due to the violation of normality, we primarily used Wilcoxon signed-rank tests to compare the pre- and post-treatment raw scores of the AAT subtests and the profile level. Since the spontaneous speech subscales are ordinal scales, we used Wilcoxon signed-rank tests without discarding zero differences. The *p* values were corrected with the Bonferroni–Holm method [29] for 12 tests (five subtests, the profile level and six spontaneous speech subscales). We calculated the matched-pairs rank biserial correlation coefficient *r*_*c*_ as effect sizes for the Wilcoxon signed-rank tests according to King et al. [30]. An effect size of ≥ 0.1 was considered as small, ≥ 0.3 as medium and ≥ 0.5 as large [31]. The ‘*coin*’ (v.1.4.2) and ‘*rcompanion*’ (v.2.4.18) packages in R were used for the Wilcoxon signed-rank tests and the effect sizes *r*_c_. One-tailed tests were carried out for all comparisons between pre- and post-treatment AAT on the group and individual level because we expected improvements after treatment. Results are reported for observed, unadjusted scores and for scores after correction for spontaneous recovery with the individual PWA-oriented approach and the mean change scores of the historical control group.

For the group-oriented approach of taking the spontaneous recovery into account, we calculated standardized mean differences (unbiased Cohen’s *d*) for each group of aphasia syndrome by subtracting changes of the historical control group from changes of the cohort of this study (i.e., therapy–control). We report the average standardized mean difference of all aphasia syndrome groups and test if it differs significantly from zero. The following adjustments were applied for calculating the standardized mean differences. First, we used the number of PWA and the frequency distributions of aphasia syndromes of this therapy study to calculate weighted standardized mean differences for the historical control group. Second, we divided the mean raw change scores between the AAT assessments and their standard derivation in the historical control group by two. This adjustment increased the comparability of the two cohorts, since PWA in the control group were assessed at fixed time points with a time interval of 3 months (1, 4 and 7 months post-onset) while PWA in this study were assessed with an interval of 6–7 weeks at variable time points post-onset. We report *p* values from random effects models using the meta-analysis R package *‘metaphor’* (v.1.2.4) implemented in jamovi [32].

For the secondary objectives, we conducted a repeated measures ANOVA for the AAT profile level as an overall measure of aphasia severity with time (pre- and post-treatment) as a within-subject factor and group of chronicity (early subacute, late subacute and chronic group) as a between-subject factor. Since data violated the assumptions of normality and homoscedasticity, we used Wilcox’s robust ANOVA for mixed designs [33] implemented in the ‘*WRS2*’ (v.1.1.5) package in R. In addition, the relationship of four quantitative (age at onset, time post-onset, dosage of SLT and aphasia severity) and two categorical predictor variables (sex, aphasia syndrome) with the binary outcome responder/nonresponder was investigated by employing multivariable logistic regression. The rationale for selection of the variables to be included in the multivariable model was based on current knowledge of the factors which may potentially influence treatment response in PWA. In the logistic regression, we accounted for possible nonlinear relationships between the continuous variables and therapy outcome by using restricted cubic splines with three knots. We also examined possible interactions with age, dosage of SLT and time post-onset with an overall interaction test [34]. Wald’s statistic was used to check for linearity and additivity. Odds ratios (OR) are reported for all continuous variables. The ‘*rms*’ package (v.6.2.0) in R was used for modelling. These secondary analyses are based on scores and therapy responders after applying the individual PWA-oriented approach to correct for spontaneous recovery using the mean change scores of the historical control group.

The threshold for significance was set to *p* = 0.05 for all tests. Statistical analyses were carried out with jamovi v.2.5 (The jamovi project, 2024, https://www.jamovi.org) and R studio 2023.06.2 (RStudio Team, 2023. RStudio: Integrated Development for R, http://www.rstudio.com/) with R v.4.1.2 (R Core Team, 2021. R: A language and environment for statistical computing, https://www.R-project.org).

Due to the real-world setting and the nature of the intervention, no blinding with respect to treatment exposure could be implemented in this study design. A randomization to no treatment or sham treatment is not acceptable ethically in a real-world setting. Given the evidence on the spontaneous course of aphasia in the chronic phase (i.e., no further improvements without intervention), we believe a pre- and posttest design to be sufficiently appropriate.

## Results

**Fig. 1:**
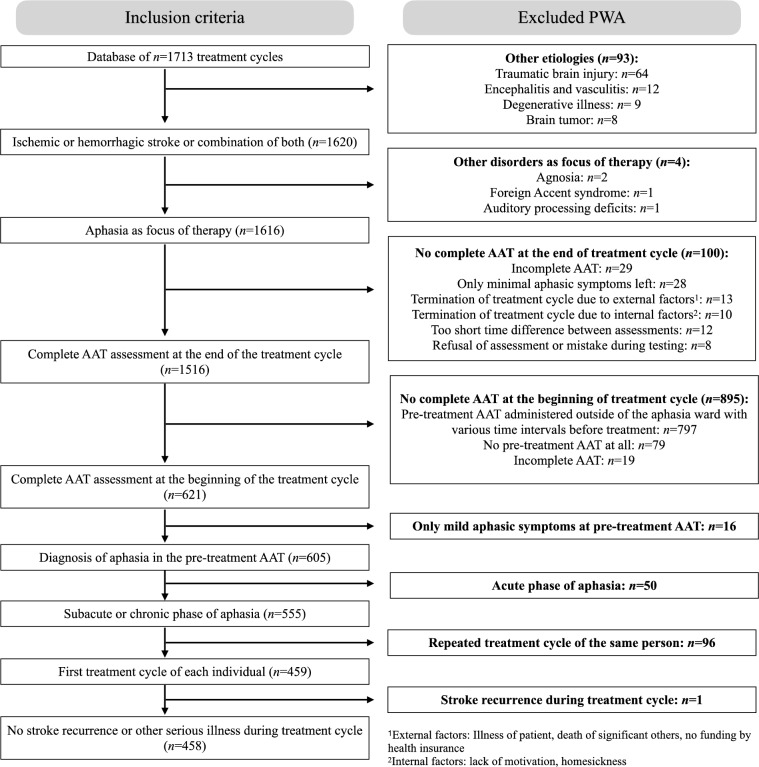
Inclusion criteria and number of excluded patients. Final sample size of *n* = 458 is the number of patients before exclusion of extreme outliers (*n* = 10). External factors refer to reasons for termination of treatment cycle which were not under control of the patient, e.g., illness, death of relatives or no funding by health insurance. Internal factors include lack of motivation or homesickness, among others

A total of 1713 treatment cycles were applied to PWA including instances of repeated treatment of the same person. After applying the inclusion criteria of this study and removing extreme outliers, the final sample size comprised *n* = 448 PWA with one treatment cycle each. Figure 1 shows that the most frequent reason for exclusion of treatment cycles was the administration of the pre-treatment AAT outside of the aphasia ward with varying time intervals before the start of treatment. These pre-treatment AATs were sufficient at the time for clinical decisions but were excluded from this study for reasons of scientific rigor.

In the remaining cohort, 52 PWA (12%) were in the early subacute, 65 PWA (15%) in the late subacute and 331 PWA (74%) in the chronic phase of aphasia. Table 2 shows the baseline characteristics at pre-treatment assessment.

**Table 2:** Baseline characteristics of entire cohort and results of univariate analyses for responders vs. nonresponders

	Entire cohort (*n* = 448)	Responders^a^ (*n* = 265)	Nonresponders^a^ (*n* = 183)	*p* value	Effect size (95% CI)
Demographics					
Age at onset (years)	53 (44–61)	51 (43–59)	54 (45–63)	0.046*	0.11 (0.00–0.22)
Female sex	130 (29)	77 (29)	53 (29)	0.983	-
Handedness^b^					
Right	217 (76)	123 (75)	94 (78)	0.811	-
Left	3 (1)	2 (1)	1 (1)		
Ambidextrous	66 (23)	40 (24)	26 (21)		
Education level^c^					
High^d^	155 (50)	86 (48)	69 (53)	0.325	-
Middle^e^	81 (26)	46 (26)	35 (27)		
Low^f^	73 (24)	48 (27)	25 (19)		
Stroke characteristics					
Time post-onset (months)	11 (6–20)	11 (6–19)	11 (6–23)	0.268	-
Stroke subtype					
Ischemic	365 (81)	217 (82)	148 (81)	0.963	-
Hemorrhagic	71 (16)	41 (16)	30 (16)		
Ischemic and hemorrhagic	12 (3)	7 (3)	5 (3)		
Stroke localization					
Left hemisphere	431 (96)	254 (96)	177 (97)	0.276	-
Right hemisphere	10 (2)	8 (3)	2 (1)		
Both hemispheres	7 (2)	3 (1)	4 (2)		
Aphasia characteristics					
Aphasia syndrome					
Global	161 (36)	87 (33)	74 (40)	0.166	-
Broca	100 (22)	61 (23)	39 (21)		
Wernicke	69 (15)	37 (14)	32 (18)		
Anomic	33 (7)	23 (9)	10 (6)		
Not classified^g^	85 (19)	57 (22)	28 (15)		
Aphasia severity^h^					
Mild	79 (18)	52 (20)	27 (15)	0.280	-
Moderate	153 (34)	93 (35)	61 (33)		
Severe	216 (48)	120 (45)	95 (52)		
Activities of daily living					
Barthel Index^i^	100 (85–100)	100 (90–100)	95 (80–100)	0.014*	0.13 (0.03–0.23)

Values shown as median (interquartile range) or numbers (proportion). Proportion may not sum up to 100 due to rounding

Univariate analyses with *χ*^2^ test and Mann–Whitney *U* test, as appropriate

*CI* confidence interval

^a^Responders and nonresponders after individual correction for spontaneous recovery with the mean change scores of the historical control group

^b^Data on handedness were missing for 36% of the entire cohort, 38% of responders and 34% of nonresponders

^c^Data on education level were missing for 31% of the entire cohort, 32% of responders and 30% of nonresponders

^d^German upper secondary education (= 12 or 13 years of school) or Bachelor’s or equivalent or Master’s or equivalent or PhD or equivalent

^e^German lower secondary education “Realschule” (= 10 years of school)

^f^German lower secondary education “Hauptschule” (= 9 years of school) or school for special needs or no school-leaving qualification after the primary education

^g^Including conduction and transcortical aphasia

^h^Determined according to the height of the AAT profile level (*T* score metric: mild ≥ 55; moderate = 46–54; severe < 46)

^i^Barthel Index = score between 0–100, which measures performance in activities of daily living, e.g., feeding and toileting

^*^p < 0.05

### Therapy outcome on the group level

The average time difference between pre- and post-treatment AAT in this study was 43 days (42–44). Wilcoxon signed-rank tests showed improvements between pre- and post-treatment for all subtests and the profile level for the unadjusted, observed raw scores as well as for the raw scores individually corrected for spontaneous recovery. We found large effect sizes for the profile level and for the subtests ‘Naming’, ‘Repetition’ and ‘Written Language’. The subtest ‘Comprehension’ showed medium effect sizes. The smallest effect size was found for the subtest ‘Token Test’ (Table 3). Parametric tests (paired *t*-tests) showed the same results with similar effect sizes (Supplementary Table 6).

**Table 3:** Comparison of pre- and post-treatment AAT subtests and profile level with raw scores after correction for spontaneous recovery and unadjusted raw scores

	Raw scores corrected for spontaneous recovery			Unadjusted raw scores		
	*z* value	*p* value	Effect size (95% CI)	*z* value	*p* value	Effect size (95% CI)
Subtests and profile level						
Token test	4.75	< 0.001*	0.22 (0.11–0.32)	4.40	< 0.001*	0.27 (0.17–0.37)
Repetition	11.53	< 0.001*	(0.55–0.71)	12.30	< 0.001*	0.64 (0.60–0.75)
Written language	9.46	< 0.001*	0.55 (0.47–0.63)	10.91	< 0.001*	0.61 (0.53–0.69)
Naming	11.51	< 0.001*	0.66 (0.58–0.73)	12.23	< 0.001*	0.69 (0.62–0.75)
Comprehension	7.77	< 0.001*	0.43 (0.33–0.53)	8.70	< 0.001*	0.48 (0.39–0.57)
Profile level	13.42	< 0.001*	0.74 (0.66–0.80)	14.69	< 0.001*	0.80 (0.74–0.85)
Spontaneous speech subscales						
Communicative behavior^a^	–	–	–	9.06	< 0.001*	0.25 (0.19–0.30)
Automatized language^a^	–	–	–	5.30	< 0.001*	0.22 (0.13–0.30)
Articulation and prosody^a^	–	–	–	10.04	< 0.001*	0.17 (0.11–0.23)
Semantic structure^a^	–	–	–	7.36	< 0.001*	0.26 (0.20–0.32)
Phonologic structure^a^	–	–	–	8.10	< 0.001*	0.21 (0.13–0.28)
Syntactic structure^a^	–	–	–	9.44	< 0.001*	0.20 (0.15–0.26)

Comparisons with non-parametric Wilcoxon signed-rank tests, one-tailed. Test statistics and effect sizes (matched-pairs rank biserial correlation coefficients *r*_*c*_) calculated according to King et al. [30]. CI: confidence interval

^a^No correction for spontaneous recovery for the spontaneous speech subscales

^*^p < 0.001

In addition, all spontaneous speech subscales improved after therapy on the group level. There was a high percentage of PWA with no difference between the pre- and post-treatment rating in all spontaneous speech subscales (71–86%) due to the restricted scale length of the 6-point rating scales. Thus, the median of all rating subscales was identical at pre- and post-treatment AAT and the effect sizes were small when not discarding the zero differences in the Wilcoxon signed-rank test (Table 3).

### Comparison with the historical control group

The average standardized mean difference in change (therapy–control) was significantly larger than zero only in the subtest ‘Repetition’ for the early subacute phase. For the other subtests and the profile level, the average standardized mean differences were only slightly above or even below zero (Table 4). For the late subacute phase, the average standardized mean difference was significantly larger than zero for the subtests ‘Repetition’ and ‘Naming’. The value of the AAT profile level was numerically comparably high but not significantly so. There was a significant heterogeneity of the true effect sizes across the aphasia syndromes in the subtest ‘Written language’ and the AAT profile level with smaller effect sizes for PWA with Global and Wernicke aphasia (Table 4).

**Table 4:** Analysis of standardized mean difference in change of AAT performance between the therapy cohort in this study and the historical control group separately for the early and late post-acute phase

	Early subacute phase		Late subacute phase	
	Average standardized mean difference^a^ (95% CI) (therapy–control)	*p* value	Average standardized mean difference^a^ (95% CI) (therapy–control)	*p* value
Token test	−-0.27 (−0.66–0.12)	0.174	−0.15 (−0.50–0.20)	0.397
Repetition	**0.43** (0.04–0.82)	0.032*	**0.78** (0.42–1.14)	< 0.001**
Written language	−0.15 (−0.62–0.32)	0.530	0.32 (−0.27–0.91)	0.283^b^
Naming	0.05 (−0.38–0.48)	0.832	**0.56** (0.07–1.05)	0.025*
Comprehension	−0.36 (−0.75–0.03)	0.073	*0.30* (−0.05–0.64)	0.095
Profile level	0.02 (−0.40–0.45)	0.910	*0.53* (−0.08–1.14)	0.088^b^

Test of difference between this therapy study and the historical control group concerning the standardized mean difference (unbiased Cohen’s *d*) in change scores of performance, utilizing a random effect model [32]. The individual study factor considered was aphasia syndrome group. Sample sizes of the control group were assumed to be identical to those in this study, and control parameter estimates were adjusted for approximately only half the phase duration in this therapy study. In bold (*italics*): estimates of average standardized mean difference along with 95% confidence intervals, with values completely (or *almost completely*) above zero. For all outcome measures, there was no indication of an outlier group or an overly influential group

*CI* confidence interval

^a^Standardized mean difference is the unbiased Cohen’s *d*

^b^Significant heterogeneity of true effect size across syndrome groups (smaller effect sizes for PWA with Global and Wernicke aphasia)

^*^p < 0.05

^**^p < 0.001

Another approach to investigate therapy-induced improvements in the early and late subacute phases confirms that language gains in the subtests ‘Repetition’ and ‘Naming’ likely go beyond spontaneous recovery in this study (see Supplemental methods and Supplementary Table 7).

### Therapy outcomes on the individual level

Table 5 shows the number of therapy responders before and after correction for spontaneous recovery. The responder rate was 63% using a ‘liberal’ correction and did not fall beyond 57% using a ‘conservative’ correction for the entire cohort. After correction for spontaneous recovery with the historical mean change score, 59% PWA showed significant improvements between pre- and post-treatment AAT, with the highest responder rate being in the early subacute group. In these 265 instances of fulfilling the responder definition, in 99 cases (37%) significant improvement was reflected in one, in 89 cases (34%) in two and in 77 cases (29%) in three or more subtests, profile level or spontaneous speech subscales. Responders after individual correction for spontaneous recovery were about 3 years younger than nonresponders (*z* = 2, *p* = 0.046) and had a higher Barthel index (*z* = −2.46,* p* = 0.014) (Table 2), received more hours of SLT (*z* = −3.21, *p* = 0.001) and more computer-based SLT (*z* = −2.85, *p* = 0.004) (Table 1). However, effect sizes of these differences were small with *r*_c_ < 0.2.

**Table 5:** Treatment response in the different subgroups before and after correction for spontaneous recovery

Group	Early subacute^a^	Late subacute^b^	Chronic^c^	Entire cohort
*n*	52	65	331	448
Responders before correction for spontaneous recovery, n (%)	48 (92%)	49 (75%)	198 (60%)	295 (66%)
Responders after average correction for spontaneous recovery, n (%)	32 (62%)	35 (54%)	No correction	265 (59%)
Responders after ‘liberal’ correction for spontaneous recovery, n (%)	40 (77%)	45 (69%)	No correction	283 (63%)
Responders after ‘conservative’ correction for spontaneous recovery, n (%)	28 (54%)	30 (46%)	No correction	256 (57%)

Mean raw score differences between AAT assessments of the historical control group were used for average correction, Q1 was used for liberal correction and Q3 was used for conservative correction for spontaneous recovery

Q1: First quartile, Q3: Third quartile

^a^6 weeks to 3 months post-onset

^b^4 to 6 months post-onset

^c^ ≥ 7 months post-onset

### Differential therapy effects

**Fig. 2:**
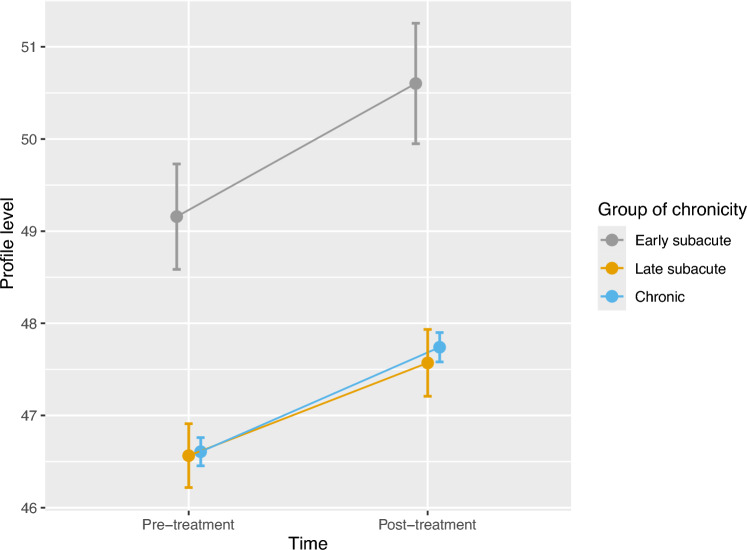
Differential effects between groups of chronicity for the AAT profile level after individual correction for spontaneous recovery. Mean AAT profile level from pre-treatment to post-treatment assessment in the early subacute, late subacute and chronic group. Error bars represent 95% CI for the variation in intraindividual changes [35]

The amount of improvements in the AAT profile level between pre- and post-treatment did not depend on the group of chronicity (*F*(2,49.11) = 0.04, *p* = 0.963) after individual correction for spontaneous recovery. Figure 2 visualizes parallel improvements for the early subacute, late subacute and chronic group after correction for spontaneous recovery. The patients showed higher profile levels after therapy (*F*(1,56.98) = 51.60, *p* < 0.001) and the profile level did not differ between the groups (*F*(2,50.32) = 2.90, *p* = 0.064). The 95% confidence interval (error bars in Fig. 2) for the variation in intraindividual changes is widest for the early subacute group and becomes narrower with time post-onset. This is in line with the correlations between the assessments in the historical control group, pointing in the direction of more variability in the changes in the earlier phases of recovery. Similar results were shown in the robust mixed ANOVAs for each of the AAT subtests (see Supplementary Table 8 and Supplementary Figs. 1–5).

### Factors associated with therapy outcome

**Fig. 3:**
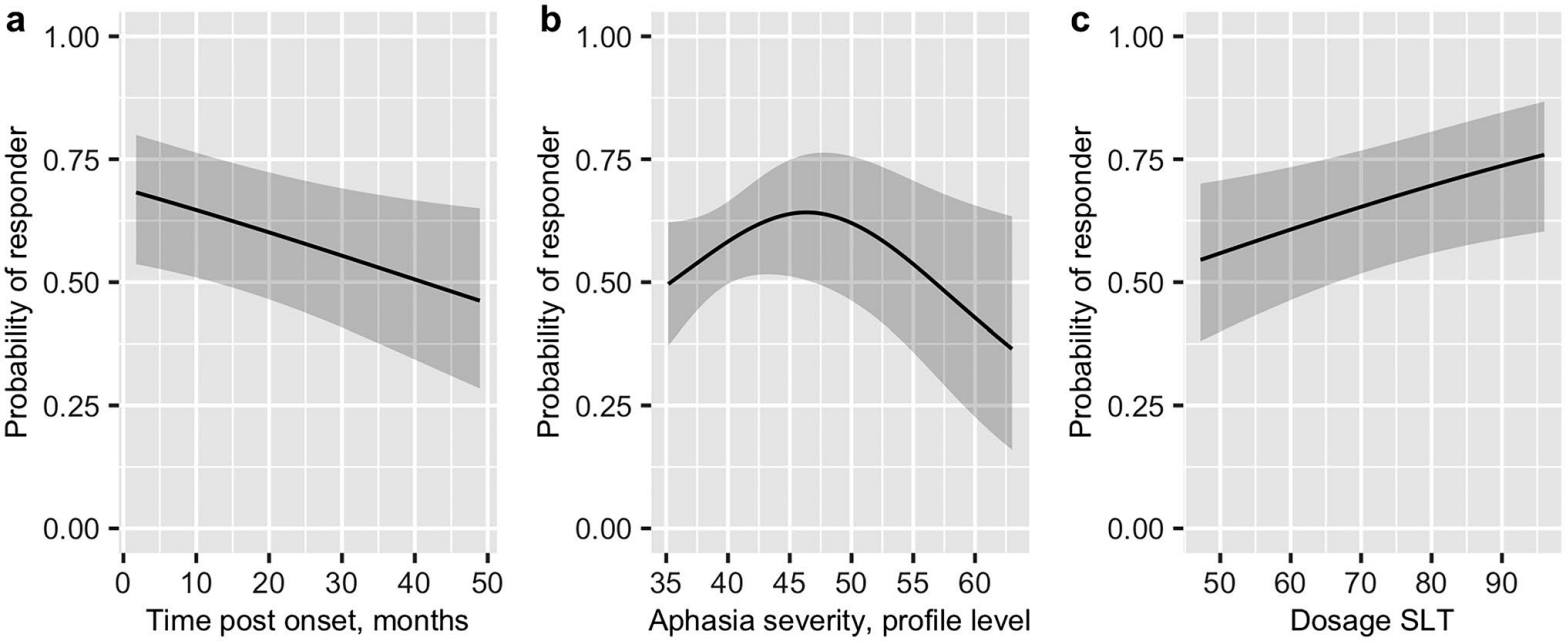
Relationship between the statistically significant variables of the logistic regression model and the probability of being a responder.** a** Time post-onset in months. **b** Aphasia severity expressed in the AAT profile level. **c** Dosage of SLT. The partial effect plots show the relationship between the plotted variables while holding the other variables constant to their median for continuous and mode for categorical variables (Sex = male, Age at onset = 53 years, Time post-onset = 11 months, Aphasia syndrome = Global, Aphasia severity = 46 *T* scores, Dosage SLT = 68 h). Responders are PWA with significant improvements after individual correction for spontaneous recovery with the mean change score from the historical control group. Grey areas present 95% CI

Tests for linearity revealed a nonlinear relationship between aphasia severity and the probability of being a responder after individual correction for spontaneous recovery (Wald *p* = 0.028). In the logistic regression model allowing for nonlinearity, the probability of being an immediate therapy responder decreases with longer duration of aphasia and increases with more hours of SLT (Table 6). The PWA with AAT profile levels at the extreme ends (i.e., very severe and very mild aphasia) had a lower probability of being a responder (Fig. 3). The overall interaction tests for interaction with age (*p* = 0.219), interaction with dosage of SLT (*p* = 0.177), and interaction with time post-onset (*p* = 0.487) were not statistically significant.

**Table 6:** Results of the logistic regression model with 3-knots restricted cubic spline for aphasia severity

Variable	OR	95% CI	*p* value
Sex (female vs male)	0.964	0.622–1.494	0.869
Age, per year (IQR = 16.62)	0.822	0.624–1.084	0.165
Time post-onset, per month (IQR = 13.86)	0.765	0.600–0.973	0.03*
Aphasia syndrome			
Global			
Broca	1.144	0.547–2.386	0.721
Wernicke	0.745	0.366–1.516	0.417
Anomic	3.153	0.915–10.971	0.069
Not classified^a^	1.618	0.774–3.382	0.200
Dosage SLT, per hour (IQR = 15.58)	1.361	1.046–1.771	0.022*
Aphasia severity^b^, per *T* score (IQR = 11.9)	0.970	0.572–1.647^c^	0.024*

Odds ratios are calculated for interquartile range effects for continuous variables

*OR* Odds ratio, *CI* confidence interval, *IQR* interquartile range

^a^Including conduction and transcortical aphasia

^b^Expressed in AAT profile level at pre-treatment assessment

^c^95% CI of OR crossing 1 because of nonlinear term

^*^p < 0.05

### Long-term stability

Follow-up data for our additional exploratory analysis were available for *n* = 70 PWA with a median of 12 months (IQR = 9–18) between post-treatment and follow-up assessment. Of these patients, 52 (74%) were immediate responders to the intensive treatment cycle under investigation. On the individual level, 12 PWA (23%) of these immediate responders had no significant changes between post-treatment and follow-up AAT, 3 (6%) had significant deterioration in one of the subtests, subscales or profile level, 33 (64%) had significant further improvements and 4 (8%) had deterioration in one and improvement in another subtest, subscale or profile level when comparing to the AAT at discharge.

## Discussion

The primary objective of our study was to investigate the outcome of a personalized, intensive SLT in a large cohort under clinical conditions in a real-world setting. Group-level analyses revealed improvements for all language modalities and spontaneous speech subscales as assessed with the AAT. Based on a large sample size, the results support previous findings showing language improvements after intensive SLT for PWA in the subacute [7–9] and chronic [11, 12, 14] phases of aphasia.

We used the AAT as the outcome measure for therapy responsiveness. This standardized language test with high reliability coefficients for its subtests [22] also allowed us to apply psychometric single-case analysis. Recent literature emphasizes the importance of critical change scores at the individual level and recommends to report the treatment response rate in any aphasia intervention study [36]. In our study, we found a high overall treatment response rate of 59% after individual correction for spontaneous recovery. As discussed below, the estimation of the spontaneous recovery without an actual control group is challenging. However, by applying both conservative and liberal estimates of spontaneous recovery from a well-characterized historical control group, we assume the ‘real’ responder rate to fall in a robustly estimated range of 57% to 63%. A previous study at the Aachen aphasia ward from 1989 with 68 PWA reported a similar overall responder rate of about 63% [8]. This is in contrast to a recent large meta-analysis of 44 trials showing individual improvements in only one third of participants [37]. However, therapy approaches, SLT dosage, outcome measures, sample sizes and responder rates varied considerably between the different studies, and only PWA with chronic aphasia were included. We argue that the individualized, intensive speech and language therapy under investigation in this study can yield responder rates that go well beyond previously reported numbers.

While the majority of the studies in the meta-analysis investigating individual improvements [37] measured the impairment-based outcome in only one specific dimension (e.g., naming), our definition of therapy success was broader, namely looking at all of the different language modalities and spontaneous speech dimensions. Hence, our definition also takes into account less often investigated, linguistic and impairment-based dimensions, e.g., comprehension, writing and reading. Here, we show improvements in the different dimensions with no exceptions and substantial effect sizes for several domains. Improvement in these modalities might also have a high impact on the individual person and be particularly important for returning to work and improving quality of life. However, we can only draw limited conclusions about the effectiveness on everyday communication skills or quality of life. Future work should include these outcome measures as well.

Given this broader definition of a responder in our study, one might have expected even higher responder rates. Although the AAT is a comprehensive diagnostic tool, it cannot measure all possible improvements after a personalized aphasia therapy as it is usually conducted under real-world conditions. Specifically, improvements in therapy targets of PWA with very severe or very mild aphasia (e.g., alternative communication and word-finding tasks beyond picture naming) are not covered by the AAT. In addition, distortions in score distributions at the extreme ends of the AAT make it much harder to detect significant improvements in PWA with very poor or very good performance [22]. In order to capture improvements covering the whole range of aphasia severity, additional and more specific measurement tools are needed. Thus, we interpret the responder rate of 59% in this study as substantially high, given the large number of PWA with a variety of aphasia syndromes and severities and the individualized therapy which targets more than the linguistic abilities covered by the AAT.

The large sample size in this study gave the opportunity to divide our cohort into sizeable subgroups of chronicity and, thus, to study differential therapy effects. The groups of early subacute, late subacute and chronic aphasia did not differ in their degree of therapy-induced improvement after individually correcting for spontaneous recovery. All subgroups showed significant improvements in all AAT subtests and the profile level. In addition, these subgroups together with the data from a historical control group [23] facilitated an investigation of therapy effects specifically in the early and late subacute phases of aphasia. In summary, we found the strongest indications for language gains beyond spontaneous recovery for the subtests ‘Repetition’ and ‘Naming’. In the early subacute phase, the language gains of PWA in this study did not exceed the estimated spontaneous recovery for the majority of subtests and the profile level. It should be noted, that PWA in this study started their intensive treatment cycle 3 months post-onset in average while PWA in the historical control group were already assessed 1 month post-onset for the first time. Assuming a nonlinear course of spontaneous recovery in the first 3–4 months after stroke with more language recovery at the beginning [38, 39], the PWA of the historical control group might just have shown more language gains because they were assessed about 2 months earlier in their recovery as compared to our cohort. Dividing the change scores of the historical control group by two might not outweigh these differences in time points of assessments and, thus, the treatment effects for PWA of the early subacute phase in our study might be underestimated. Of course, our approaches to compare this cohort with the historical control group and to individually correct for spontaneous recovery cannot completely compensate for the large heterogeneity of the entire population of PWA. Therefore, the results regarding genuine therapy effects in the early and late subacute phase of aphasia need to be interpreted with some caution. Still, with our primary goal of investigating therapy outcomes of intensive SLT under clinical conditions, these approaches were the best alternatives to a matched control group without treatment. Also, having a control group receiving no therapy specifically in the subacute phase would raise ethical challenges and might be subject to substantial selection bias [8].

Beyond showing the positive immediate therapy outcome, we could also report exploratory findings about the maintenance of language gains for a subsample. On the individual level, the majority of immediate therapy responders (85%) could maintain their language gains or even improve more between post-treatment and follow-up AAT. These results should be interpreted with caution because the length of the follow-up period varied substantially and we do not have any detailed information about SLT intensity or extra access to community-based aphasia programs during that time. Still, these data indicate the long-term stability of treatment effects in a real-world setting, similar to the 6-month follow-up data of previous studies [11, 28]. Assuming a less intensive treatment period between post-treatment and follow-up assessment, the percentage of even more improvements at the individual level was surprisingly high (63%). Similar results of continued improvements on the group level during 6-month follow-up could be observed in high intensive upper limb neurorehabilitation in chronic stroke patients [40]. The authors discussed the content of the intervention including education and self-efficacy as factors contributing to further improvements after the treatment had stopped. In this study, similar ingredients of the comprehensive therapy program (e.g., communicative-pragmatic strategies, counseling of patients and their caregivers) in addition to the language improvements after therapy might lead to more participation and communication in everyday situations at home and, in turn, elicit even more improvements. This self-reinforcing effect could further be boosted by therapy-induced neuroplasticity after intensive SLT, which has an effect beyond the intensive therapy cycle. However, we must also acknowledge that PWA who come back to the aphasia ward for a second treatment cycle of intensive SLT underlie a certain selection bias because they are comparably highly motivated. All these factors need to be explored in future studies.

Recent literature on predictors of therapy response still fails to give a robust answer to the question who will respond to therapy or not [41]. Our results support the assumption that it is mainly the dosage of SLT that influences the outcome of therapy. This finding based on real-world data from routine clinical care complement previous randomized-controlled trials [11, 12] and meta-analyses [13–15] which indicate the superiority of high dosage SLT. However, dosages of SLT in these studies vary considerably (30–208 h) and the minimum dosage to achieve clinically meaningful language gains is still unclear. Although experimental studies and real-world data taken together point in the direction that intensive SLT can lead to meaningful language gains in a short amount of time, the implementation of sustainable treatment programs remains challenging. Delivery approaches other than traditional face-to-face SLT need to be considered in order to increase the dosage of therapy. Since studies indicate that alternative approaches such as remote treatment [42], communication training of caregivers [43], technology-based self-managed training [44, 45] or therapist-trained nonprofessional SLT [14] could be beneficial as well, these possibilities need to be considered as useful addition to face-to-face therapy when high intensive SLT cannot be offered.

Time post-onset was another predictor in our logistic regression analysis. However, OR reported in our study refer to an increase of almost 14 months in time post-onset (Table 4) and wide confidence intervals preclude from individual prognosis throughout the temporal range (Fig. 3a). Furthermore, high responder rates in the chronic subgroup (60%) show that improvement at the individual level is still possible months to even years after stroke. The careful interpretation of time post-onset as a predictor of therapy outcome is in line with a recent review which states that time post-onset does not seem to be related to therapy response beyond spontaneous recovery, specifically not in the chronic phase of aphasia [41]. Therefore, high chronicity alone should not be considered as an exclusion criterion when evaluating PWA for intensive aphasia therapy. As an additional, but crucial finding of our secondary analyses, we show that there is no interaction between several of the examined factors. This means for example that being both older and having suffered from a more severe stroke does not confer disproportionate disadvantages, or put another way: these PWA, too, should not be excluded from intensive therapy programmes according to our data.

There are some limitations in this study. First, selection bias is likely to be present which is mainly driven by the necessity of being capable to follow an intensive treatment regimen. Thus, PWA in this study are relatively young, rather independent in daily living, and have a good general health condition, as compared to a general stroke population. Patients fulfilling geriatric criteria are not represented in our sample. Due to high bureaucratic obstacles in dealing with health insurances and the necessity of traveling to Aachen, there might also be a selection bias towards highly motivated PWA with supportive relatives. Second, the results of this study cannot clarify whether a distributed therapy scheme with the same dosage might be similarly or even more beneficial for some PWA, and who benefits most from which approach. Some of the nonresponders in this study might have benefited more from such a distributed SLT due to factors we are currently not aware of. More research is needed for reasonable decisions on the best approach for each individual. Third, we only included the first treatment cycle of each patient in the main analyses. Future studies need to investigate responder rates in the course of repeated treatment intervals, the underlying mechanism of maintenance or even more improvement between intensive treatment cycles, and a possible plateau of improvement.

In conclusion, our study demonstrates a number of clinically relevant points. Substantial, measurable improvements can be achieved in a routine clinical setting for a high number of heterogeneous PWA. The AAT is a notoriously conservative instrument which assesses only language-systematic capabilities and not communicative abilities. Thus, the actual impact on PWAs everyday lives is probably underestimated. Clinical experience at the Aachen aphasia ward suggests extremely high patient satisfaction but further investigation is needed, for example by employing patient-reported outcomes. Although our data suggest a particular treatment effect for PWA with moderate aphasia, effect sizes of the factor aphasia severity were small and could also be attributed to bottom and ceiling effects of the AAT. Crucially, no main effects or interactions were found with chronicity or age, supporting the notion that this type of treatment is suitable for a broad spectrum of PWA extending into time periods previously regarded as highly chronic and not amenable to therapy. Finally, AAT subtest analyses robustly demonstrate that all language domains can successfully be addressed and no linguistic capability is exempt. This is important as previous studies often only assessed “naming” as their outcome measure.

## Supplementary Information

Below is the link to the electronic supplementary material.

Supplementary file1 (PDF 5 KB)

Supplementary file2 (PDF 5 KB)

Supplementary file3 (PDF 5 KB)

Supplementary file4 (PDF 5 KB)

Supplementary file5 (PDF 5 KB)

Supplementary file6 (DOCX 112 KB)

## Data Availability

The anonymized data supporting the findings of this study are available from the corresponding author, upon reasonable request. We currently are evaluating the possibility of contributing the dataset to an international aphasia dataset led by the Collaboration of Aphasia Triallists.
