# Correction to: Success rates of intensive aphasia therapy: real-world data from 448 patients between 2003 and 2020

**DOI:** 10.1007/s00415-024-12559-y

**Published:** 2024-09-14

**Authors:** Dorothea Peitz, Beate Schumann-Werner, Katja Hussmann, João Pinho, Hong Chen, Ferdinand Binkofski, Walter Huber, Klaus Willmes, Stefan Heim, Jörg B. Schulz, Bruno Fimm, Cornelius J. Werner

**Affiliations:** 1 Department of Neurology, Medical Faculty, RWTH Aachen University, Pauwelsstrasse 30, 52074 Aachen, Germany; 2 Institute of Cognitive Neurology and Dementia Research, Otto-Von-Guericke-University, Magdeburg, Germany; 3 Section Clinical Cognitive Sciences, Department of Neurology, Medical Faculty, RWTH Aachen University, Aachen, Germany; 4 Department of Psychiatry, Psychotherapy and Psychosomatics, Medical Faculty, RWTH Aachen University, Aachen, Germany; 5 Institute of Neuroscience and Medicine (INM-1), Research Centre Jülich, Jülich, Germany; 6 JARA-BRAIN Institute Molecular Neuroscience and Neuroimaging, Research Centre Jülich GmbH, Jülich, Germany; 7 JARA-BRAIN Institute Molecular Neuroscience and Neuroimaging, RWTH Aachen University, Aachen, Germany; 8 Department of Neurology and Geriatrics, Johanniter Hospital Stendal, Hansestadt Stendal, Germany

**Correction to: Journal of Neurology** 10.1007/s00415-024-12429-7

In the original version of this article, In Fig. 3, the gray shades indicating the confidence intervals are missing.

In Table 3, for the Repetition subtest, the effect size has been lost in the article (only the 95%CI is in brackets). The effect size should be 0.64. The correct Table 3 should have been.

**Table 3:** Comparison of pre- and post-treatment AAT subtests and profile level with raw scores after correction for spontaneous recovery and unadjusted raw scores

	Raw scores corrected for spontaneous recovery			Unadjusted raw scores		
	*z* value	*p* value	Effect size (95% CI)	*z* value	*p* value	Effect size (95% CI)
Subtests and profile level						
Token test	4.75	< 0.001*	0.22 (0.11–0.32)	4.40	< 0.001*	0.27 (0.17–0.37)
Repetition	11.53	< 0.001*	(0.55–0.71)	12.30	< 0.001*	0.64 (0.60–0.75)
Written language	9.46	< 0.001*	0.55 (0.47–0.63)	10.91	< 0.001*	0.61 (0.53–0.69)
Naming	11.51	< 0.001*	0.66 (0.58–0.73)	12.23	< 0.001*	0.69 (0.62–0.75)
Comprehension	7.77	< 0.001*	0.43 (0.33–0.53)	8.70	< 0.001*	0.48 (0.39–0.57)
Profile level	13.42	< 0.001*	0.74 (0.66–0.80)	14.69	< 0.001*	0.80 (0.74–0.85)
Spontaneous speech subscales						
Communicative behavior^a^	–	–	–	9.06	< 0.001*	0.25 (0.19–0.30)
Automatized language^a^	–	–	–	5.30	< 0.001*	0.22 (0.13–0.30)
Articulation and prosody^a^	–	–	–	10.04	< 0.001*	0.17 (0.11–0.23)
Semantic structure^a^	–	–	–	7.36	< 0.001*	0.26 (0.20–0.32)
Phonologic structure^a^	–	–	–	8.10	< 0.001*	0.21 (0.13–0.28)
Syntactic structure^a^	–	–	–	9.44	< 0.001*	0.20 (0.15–0.26)

Comparisons with non-parametric Wilcoxon signed-rank tests, one-tailed. Test statistics and effect sizes (matched-pairs rank biserial correlation coefficients *r*_*c*_) calculated according to King et al. [30]. CI: confidence interval

^a^No correction for spontaneous recovery for the spontaneous speech subscales

^*^p < 0.001

The original article has been corrected.

