# Supplementary figures and images for: Success rates of intensive aphasia therapy: real-world data from 448 patients between 2003 and 2020
Source: J Neurol. 2024 May 20;271(11):7169–83. doi: 10.1007/s00415-024-12429-7 (PMC11561048; doi:10.1007/s00415-024-12429-7)

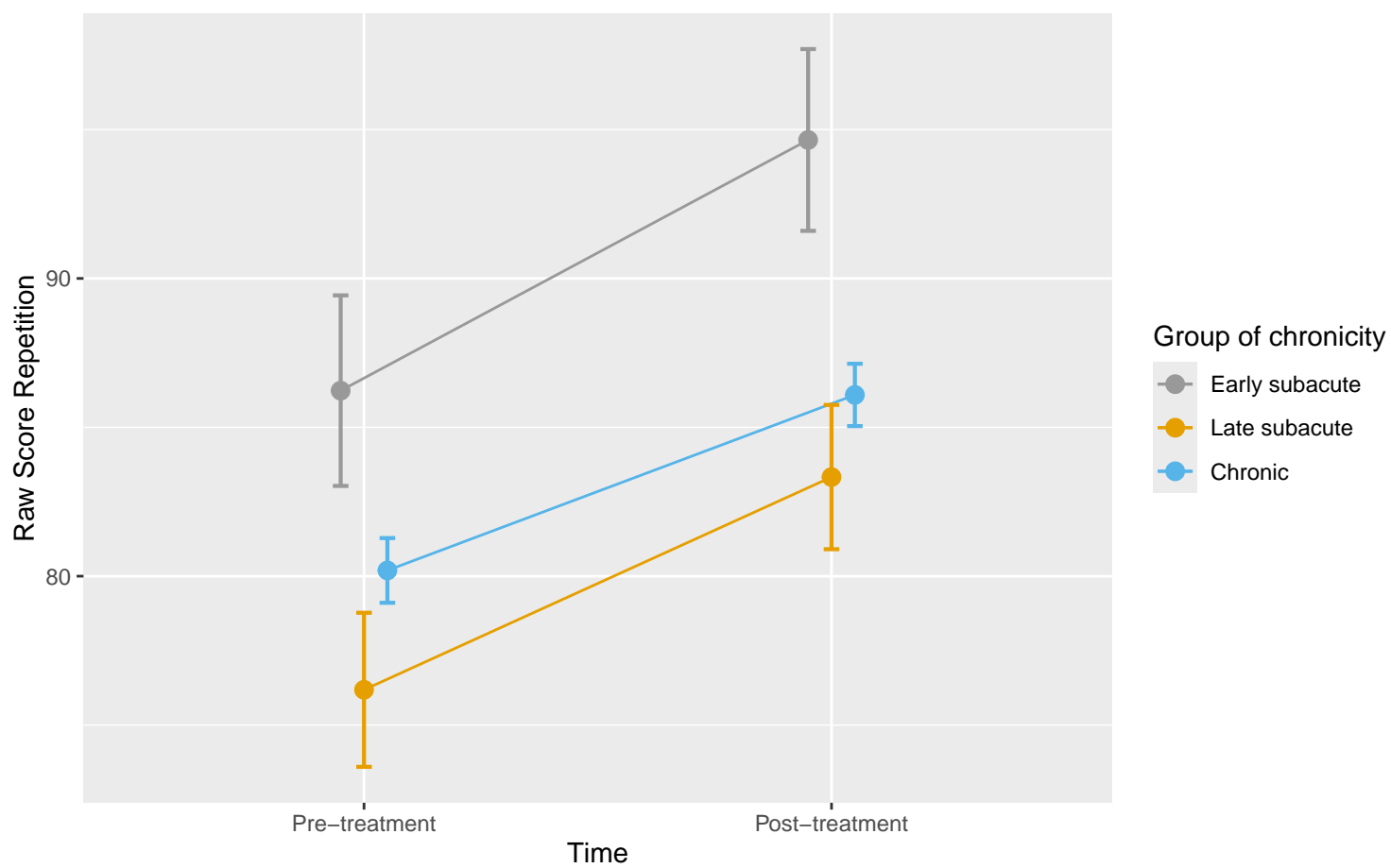

Supplement: Supplementary file 1 — Supplementary file1 (PDF 5 KB) [file 415_2024_12429_MOESM1_ESM.pdf]

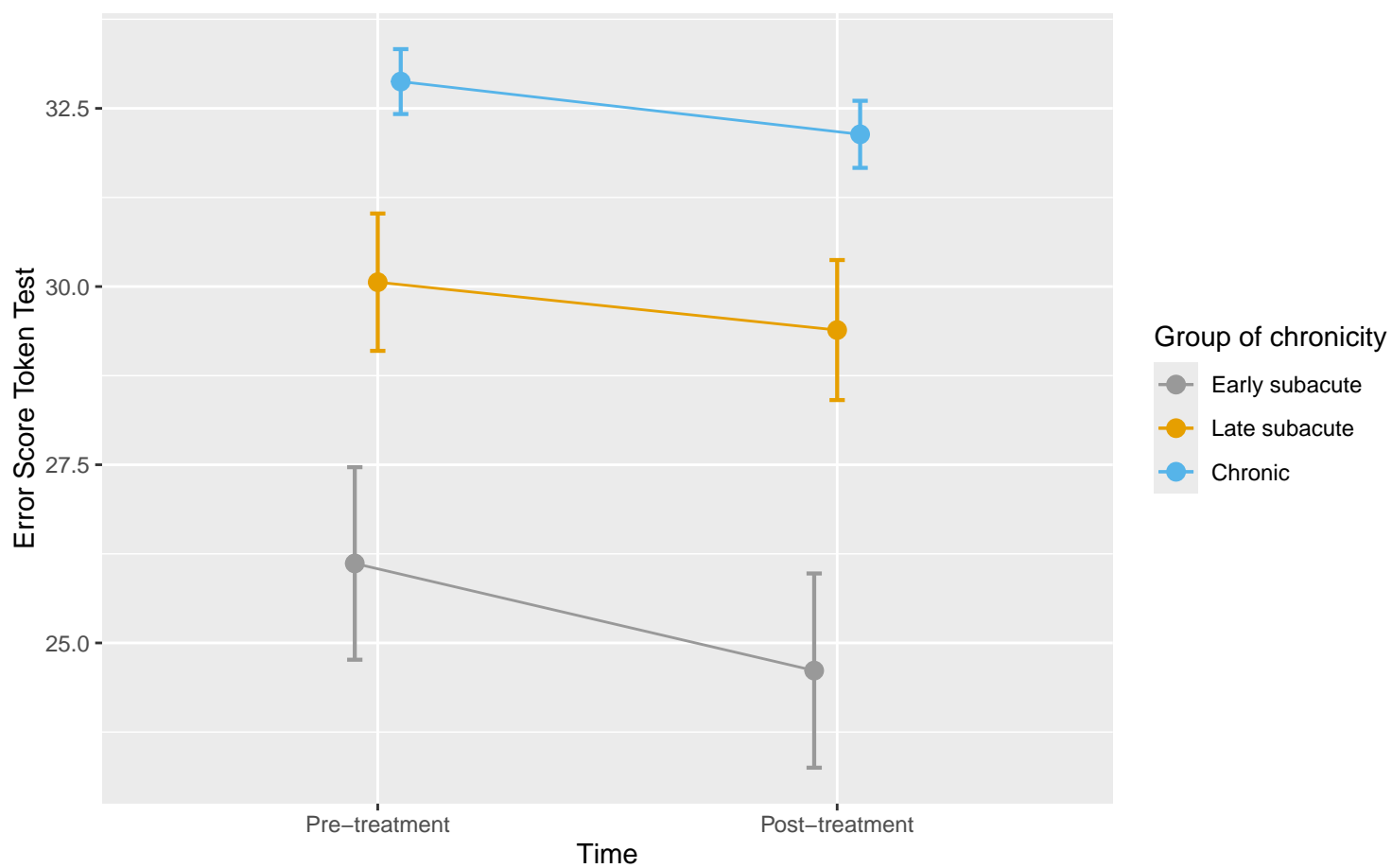

Supplement: Supplementary file 2 — Supplementary file2 (PDF 5 KB) [file 415_2024_12429_MOESM2_ESM.pdf]

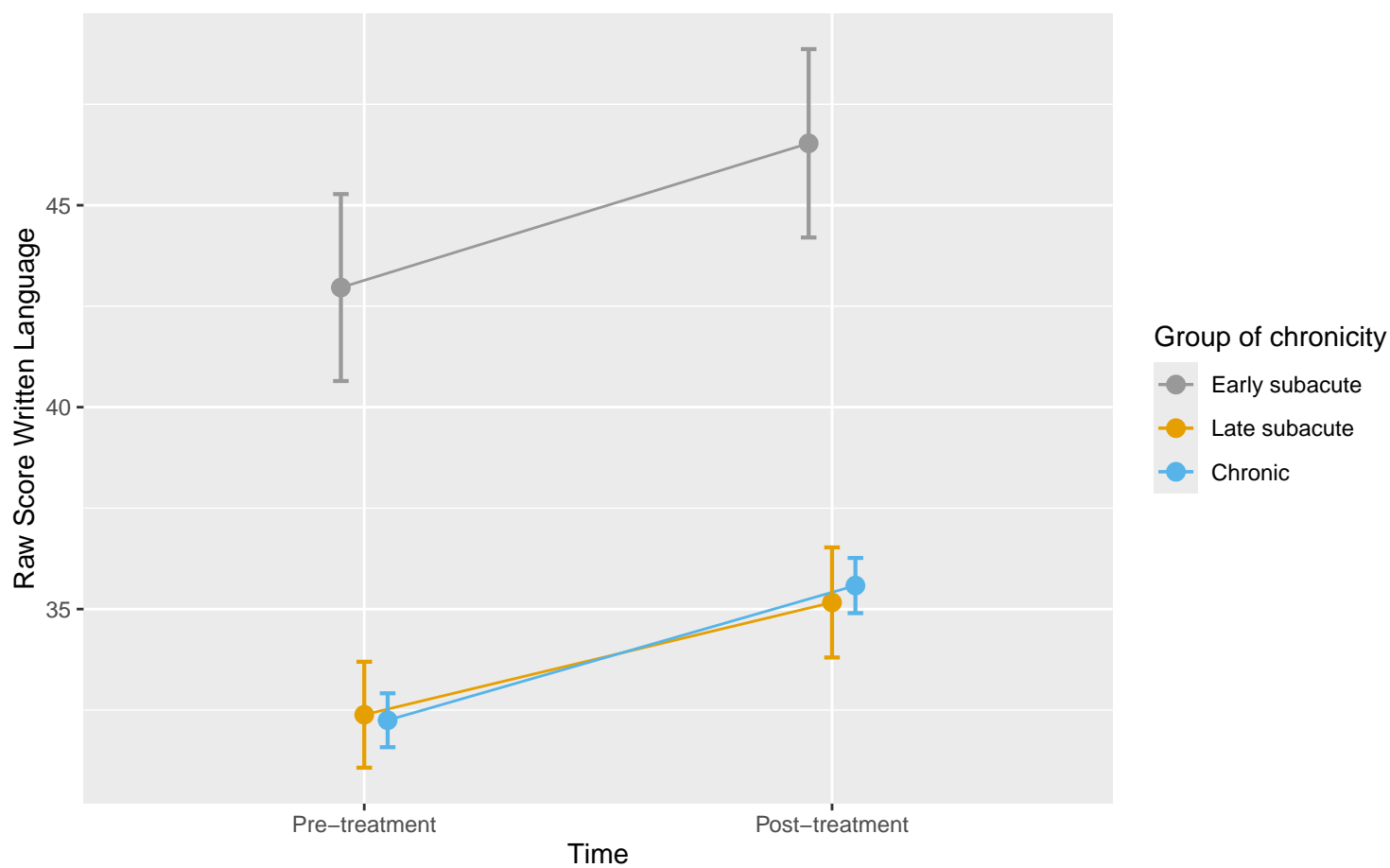

Supplement: Supplementary file 3 — Supplementary file3 (PDF 5 KB) [file 415_2024_12429_MOESM3_ESM.pdf]

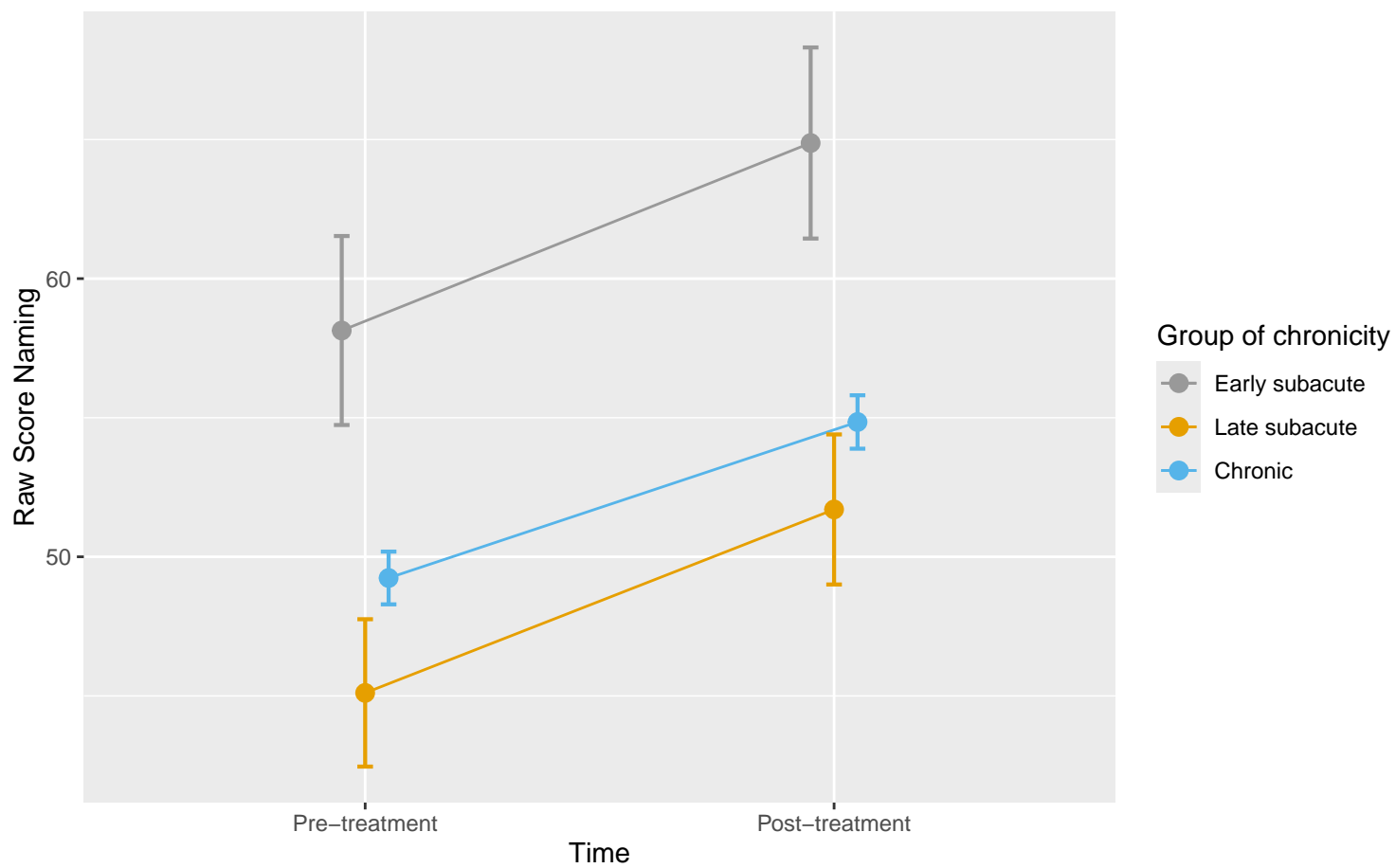

Supplement: Supplementary file 4 — Supplementary file4 (PDF 5 KB) [file 415_2024_12429_MOESM4_ESM.pdf]

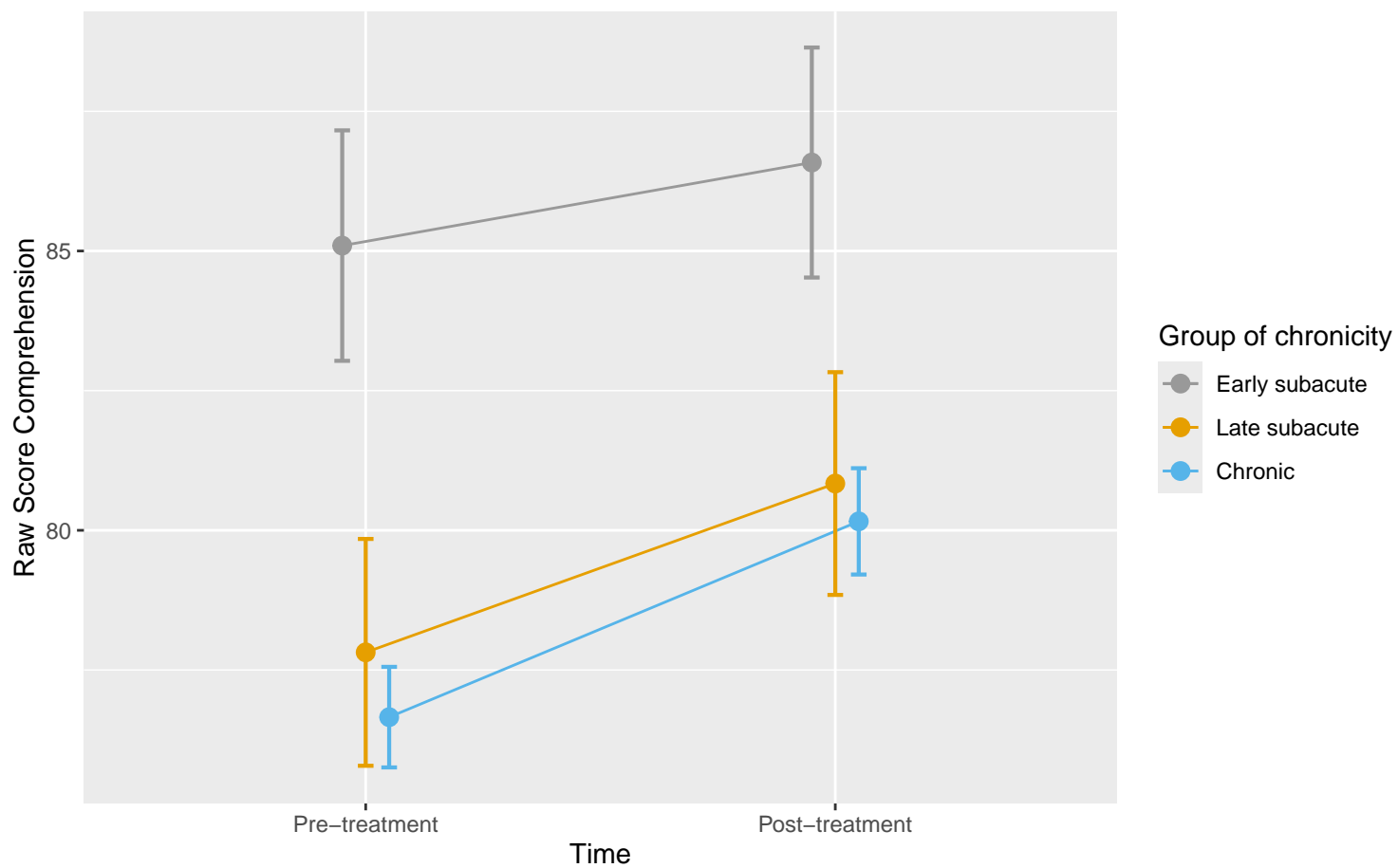

Supplement: Supplementary file 5 — Supplementary file5 (PDF 5 KB) [file 415_2024_12429_MOESM5_ESM.pdf]
