# Supplementary material for: Success rates of intensive aphasia therapy: real-world data from 448 patients between 2003 and 2020
Source: J Neurol. 2024 May 20;271(11):7169–83. doi: 10.1007/s00415-024-12429-7 (PMC11561048; doi:10.1007/s00415-024-12429-7)
Supplement: Supplementary file 6 — Supplementary file6 (DOCX 112 KB) [file 415_2024_12429_MOESM6_ESM.docx]

***Supplementary Material***

***Journal of Neurology***

Success Rates of Intensive Aphasia Therapy: Real-world data from 448 patients between 2003 and 2020

Dorothea Peitz,^1^ Beate Schumann-Werner,^1,2^ Katja Hussmann,^1^ João Pinho,^1^ Hong Chen,^3^ Ferdinand Binkofski,^3^ Walter Huber,^1^ Klaus Willmes,^1^ Stefan Heim,^1,4,5^ Jörg B. Schulz,^1,6^ Bruno Fimm^1^ and Cornelius J. Werner^1,7^

1 Department of Neurology, Medical Faculty, RWTH Aachen University, Aachen, Germany

2 Institute of Cognitive Neurology and Dementia Research, Otto-von-Guericke-University, Magdeburg, Germany

3 Section Clinical Cognitive Sciences, Department of Neurology, Medical Faculty, RWTH Aachen University, Aachen, Germany

4 Department of Psychiatry, Psychotherapy and Psychosomatics, Medical Faculty, RWTH Aachen University, Aachen, Germany

5 Institute of Neuroscience and Medicine (INM-1), Research Centre Jülich, Jülich, Germany

6 JARA-BRAIN Institute Molecular Neuroscience and Neuroimaging, Research Centre Jülich GmbH, Jülich, and RWTH Aachen University, Aachen, Germany

7 Department of Neurology and Geriatrics, Johanniter Hospital Stendal, Hansestadt Stendal, Germany

**Corresponding author:**

Cornelius J. Werner, MD

Department of Neurology, Medical Faculty, RWTH Aachen University, Pauwelsstrasse 30, 52074 Aachen, Germany

Tel.: +49 3931 66 1800, cornelius.werner@rwth-aachen.de

**Supplementary methods**

1. Outcome measures

The Aachen Aphasia Test (AAT) [1] consists of a semi-standardized interview eliciting spontaneous speech and five subtests covering all language modalities. Spontaneous speech is evaluated on six rating scales: ‘Communicative Behavior’, ‘Articulation and Prosody’, ‘Automatized Language’, ‘Semantic Structure’, ‘Phonologic Structure’, and ‘Syntactic Structure’. Each six-point rating scale ranges from 0 (most severe impairment) to 5 (no impairment). The manual of the AAT provides specific rating criteria for each of the spontaneous speech subscales’ scores. The five subtests ‘Token Test’ (50-0 errors), ‘Repetition’ (0-150 raw scores), ‘Written Language’ (0-90 raw scores), ‘Naming’ (0-120 raw scores), and ‘Comprehension’ (0-120 raw scores) consist of three to five parts of 10 items each, which test different linguistic units (phonemes, words, sentences). Except for the ‘Token Test’ with a fail/pass scoring, individual items are scored from 0 (no response or no similarity with the target) to 3 (immediate and correct response). Intermediate scores are given when responses are less or more similar to the target. Similarity criteria are specified in the test manual.

In this study, we determined language improvement in each subtest by subtracting the pre-treatment score from the post-treatment score. However, the post-treatment errors were subtracted from the pre-treatment errors for the ‘Token Test’ because improvement is reflected in less errors.

1. Individual PWA oriented correction for spontaneous recovery

In order to correct for spontaneous recovery, we used historical data from 96 people with aphasia (PWA) who had no access to speech and language therapy (SLT) in the first months after stroke [2]. These 96 PWA were assessed with the AAT at 1, 4 and 7 months post onset. Thus, the mean raw score differences of the subtests and the profile level differences between these three assessments were available, separately for the different aphasia syndromes (Supplementary Table 1). The time intervals between the AAT assessments were longer in the spontaneous recovery study (3 months) than in our study (6 weeks on average). Therefore, we interpolated the course of spontaneous recovery based on existing literature [3, 4] with a nonlinear, steeper course during 1-4 months post onset and a linear course during 4-7 months post onset. According to this interpolated course, we used multipliers smaller than (or equal to) 1 to calculate the raw score differences of spontaneous recovery for the shorter time intervals in our study (Supplementary Table 2). Depending on the aphasia syndrome and the time post onset at pre- and post-treatment assessments, the expected individually calculated raw score difference due to spontaneous recovery (correction term) was subtracted from the individually determined raw score difference of the respective patient in our study. For example, a patient with Wernicke aphasia was assessed with pre-treatment AAT at 5 months post onset and with post-treatment AAT at 7 months post onset. This patient’s raw score change was 21 in the subtest ‘Naming’ from pre- to post-treatment AAT. The following calculation was used for correction for spontaneous recovery for this patient:

$$\text{21-(0.67×4)=18.32}$$

with 0.67 as the multiplier for the AAT assessments at 5 and 7 months post onset (Supplementary Table 2) and 4 as the average change score in the subtest ‘Naming’ for the PWA with Wernicke aphasia between 4 and 7 months post onset in the spontaneous recovery study (Supplementary Table 1). Then the change score of 18.32 was compared to the critical difference of 14.71 for subtest ‘Naming’ (Supplementary Table 4). Since 18.32 exceeded 14.71, this change score is taken to indicate significant improvement in the subtest ‘Naming’ after correction for spontaneous recovery. If a patient showed no change or a deterioration between pre- and post-treatment AAT (i.e., change score ≤ 0) we used this score difference and did not correct for spontaneous recovery. If the expected raw score difference due to spontaneous recovery exceeded the observed raw score difference in our study, we determined the corrected raw score difference as 0.

Similarly, the raw score and profile level change scores were adjusted for calculating the ‘liberal’ and ‘conservative’ responder rates. The only difference in the procedure described above is that we used the first quartile (Q1) of the change scores in the spontaneous recovery study instead of the average as the lower bound of expected recovery (=liberal correction) and the third quartile (Q3) as the upper bound of expected recovery (=conservative correction). In the example of the patient with Wernicke aphasia from above, the calculation for the ‘liberal’ correction was:

$$21-\left( 0.67x1 \right)=20.33$$

with 0.67 as the multiplier for the AAT assessments at 5 and 7 months post onset (Supplementary Table 2) and 1 as the Q1 change score in the subtest ‘Naming’ for the PWA with Wernicke aphasia between 4 and 7 months post onset in the spontaneous recovery study (Supplementary Table 1). Analogous to this, the calculation for the ‘conservative’ correction was:

$$21-\left( 0.67x8 \right)=15,64$$

with 8 as the Q3 change score in the subtest ‘Naming’ for PWA with Wernicke aphasia between 4 and 7 months post onset in the spontaneous recovery group (Supplementary Table 1). If Q1 was negative we did not subtract anything from the observed change score of the PWA in our study.

1. Comparison of unadjusted language gains with the historical control group with weighted grand averages

Due the differences between the samples of this study and the historical control group and the varying time-lines of AAT assessments there are different approaches to investigate if therapy-induced improvements go beyond spontaneous recovery in the subacute phase of aphasia. Thus, the following analysis is supposed to complement the results of the main analysis in the manuscript:

The weighted grand average of the estimated spontaneous recovery in the subacute PWA (early and late subacute) of this study was calculated for each AAT subtest and the profile level separately. It expresses the expected changes due to spontaneous recovery while considering the number of PWA of each aphasia syndrome being assessed at the different time points in the course of the disease First, a weighted average for each of the aphasia syndrome (GL=Global, WE=Wernicke, BR=Broca, AN=Anomic, NC=Not Classified) was calculated:

$$\bar{D}_{syn}= \frac{\sum_{i=1}^{n} n_{i}* \bar{D}_{corri}}{\sum_{i=1}^{n} n_{i}}$$

*k* = 15 combinations of time post onset at pretreatment AAT and time post onset at posttreatment AAT which occurred in this study (see the first 15 rows of Supplementary table 2)

$\bar{D}_{corri}$ = mean change score between assessments at 1 and 4 months post onset (D_12_) or 4 and 7 months post onset (D_23_) of the group of PWA with the respective aphasia syndrome (=*syn*) in the historical control group (Supplementary Table 1), corrected with the respective multiplier for the *i*-th combination of the time post onset times from the interpolation of the course of spontaneous recovery (Supplementary Table 2, column 3)

$n_{i}$ = number of PWA for the *i*-th combination of the time post onset times in our study

We then calculated the grand average of all $\bar{D}_{syn}$ for each AAT subtest and the profile level:

$$D= \frac{n_{GL}* \bar{D}_{GL}+n_{WE}* \bar{D}_{WE}+n_{BR}* \bar{D}_{BR}+n_{AN}* \bar{D}_{AN}+n_{NC}* \bar{D}_{NC}}{n}$$

This calculation was performed separately (i) with *n* being the number of PWA in the early and late subacute phase of this study (*n* = 117) and (ii) with *n* being the number of PWA of the entire cohort (*n* = 448).

Finally, we investigated if the originally observed average raw score and profile level change scores (not corrected) in the cohort of this study are significantly larger than the expected differences caused by spontaneous recovery, using one-sample t-tests against the respective $D$ of each subtest and profile level. These tests were performed one-tailed because we expected the observed average change scores to be larger than the weighted grand averages. The analyses were performed separately for (i) only the early and the late subacute groups taken together and (ii) the entire cohort. The results are displayed in Supplementary Table 7. In summary, the most robust result of significant larger observed change scores in the subacute phases in this study than the weighted grand average can be seen in the subtests ‘Repetition’ and ‘Naming’ and in the AAT profile level, indicating therapy-induced language gains beyond spontaneous recovery in these domains.

**Table 1 AAT data from the historical control group** [2] **for correction for spontaneous recovery**

|  |  | **D_12_^a^** | | | | | **D_23_^b^** | | | | |
| --- | --- | --- | --- | --- | --- | --- | --- | --- | --- | --- | --- |
|  |  | **GL** | **WE** | **BR** | **AN** | **NC** | **GL** | **WE** | **BR** | **AN** | **NC** |
| *n* |  | 21 | 19 | 12 | 32 | 12 | 21 | 19 | 12 | 32 | 12 |
| Token Test | mean (SD) | 5  (8.3) | 10  (9.0) | 11  (5.7) | 7  (8.0) | 9  (12) | 5  (6.2) | 5  (6.0) | 1  (3.2) | 1  (3.3) | 3  (4.9) |
|  | median (IQR) | 1  (0-7) | 2  (3-16) | 12  (7-16) | 6  (2-11) | 7  (5-8) | 4  (0-9) | 5  (1-8) | 0  (-1-4) | 0  (-2-2) | 3  (0-5) |
| Repetition | mean (SD) | 37  (41.1) | 16  (20.9) | 15  (8.5) | 5  (6.2) | 11  (11.9) | 7  (8.7) | 4  (5.5) | 4  (5.0) | 1  (3.7) | 7  (9.5) |
|  | median (IQR) | 22  (6-59) | 7  (3-17) | 14  (9-20) | 4  (0-8) | 4  (3-17) | 8  (0-14) | 2  (0-6) | 3  (1-6) | 1  (-1-3) | 4  (-1-12) |
| Written Language | mean (SD) | 15  (16.1) | 25  (18.6) | 20  (15.2) | 10  (9.1) | 17  (15) | 7  (8.4) | 8  (8.5) | 0  (5.2) | 2  (3.9) | 6  (12.6) |
|  | median (IQR) | 7  (2-23) | 21  (14-34) | 18  (6-30) | 9  (2-14) | 12  (7-24) | 4  (1-13) | 5  (1-11) | 1  (-2-2) | 2  (-1-4) | 2  (-1-7) |
| Naming | mean (SD) | 31  (29.2) | 24  (13.4) | 15  (10.9) | 11  (8.3) | 19  (16.9) | 11  (13.4) | 4  (6.5) | 2  (7.3) | 2  (6.0) | 9  (17.1) |
|  | median (IQR) | 26  (7-55) | 23  (17-32) | 14  (8-21) | 9  (4-17) | 13  (6-22) | 11  (1-20) | 3  (1-8) | 1  (-5-9) | 2  (-2-5) | 5  (1-13) |
| Comprehen-sion | mean (SD) | 20  (21.9) | 21  (16.8) | 10  (8.4) | 10  (10.7) | 16  (17) | 5  (9.6) | 7  (8.8) | 1  (6.0) | 3  (5.7) | 4  (7.5) |
|  | median (IQR) | 16  (8-27) | 15  (10-29) | 12  (5-17) | 10  (4-16) | 14  (8-22) | 6  (1-11) | 5  (1-10) | 3  (-3-5) | 4  (1-7) | 7  (1-7) |
| Profile level^c^ | mean (SD) | 5.6  (5.2) | 7.1  (3.6) | 7.5  (3.6) | 5.5  (3.9) | 5.2  (3.5) | 2.4  (2.2) | 2.8  (2.5) | 1.2  (2.1) | 1.9  (2.5) | 2.6  (2.4) |
|  | median (IQR) | 2.8  (1.8-10.6) | 7.0  (4.7-9.2) | 6.8  (5.4-8.9) | 5.4  (2.9-7.5) | 4.4  (3.1-7.5) | 1.9  (1.2-3.0) | 2.6  (1.5-4.0) | 1.5  (-0.4-2.8) | 1.8  (0.1-2.8) | 2.2  (0.8-4.1) |

Mean differences (SD) and median (IQR) of AAT raw scores in the five subtests and profile level between assessments at 1, 4 and 7 months post onset of *n* = 96 PWA without treatment, separately for different aphasia syndromes.

^a^Difference between assessments at 1 and 4 months post onset.

^b^Difference between assessments at 4 and 7 months post onset.

^c^*T* score difference.

GL = Global aphasia; WE = Wernicke aphasia; BR = Broca aphasia; AN = Anomic aphasia; NC = Not classified or nonstandard aphasia syndromes.

**Table 2 Multipliers for correction for spontaneous recovery depending on the time post onset at pre- and post-treatment AAT**

|  |  |  |  | **Number of PWA** | | | | | |
| --- | --- | --- | --- | --- | --- | --- | --- | --- | --- |
|  | **Months post onset at**  **pre-treatment AAT** | **Months post onset at**  **post-treatment AAT** | **Correction term** | **GL** | **WE** | **BR** | **AN** | **NC** | **All syn** |
| Early subacute group | 1 | 2 | 0.4 x D_12_ | 0 | 0 | 0 | 0 | 0 | 0 |
|  | 1 | 3 | 0.75 x D_12_ | 0 | 0 | 0 | 1 | 0 | 1 |
|  | 1 | 4 | 1 x D_12_ | 0 | 0 | 0 | 0 | 0 | 0 |
|  | 2 | 2^a^ | 0.35 x D_12_ | 0 | 0 | 0 | 1 | 0 | 1 |
|  | 2 | 3 | 0.35 x D_12_ | 3 | 5 | 1 | 0 | 4 | 13 |
|  | 2 | 4 | 0.6 x D_12_ | 0 | 1 | 3 | 1 | 1 | 6 |
|  | 3 | 3^a^ | 0.25 x D_12_ | 0 | 0 | 1 | 0 | 0 | 1 |
|  | 3 | 4 | 0.25 x D_12_ | 2 | 5 | 3 | 2 | 6 | 18 |
|  | 3 | 5 | 0.25 x D_12_ + 0.33 × D_23_ | 6 | 2 | 1 | 1 | 2 | 12 |
| Late subacute group | 4 | 5 | 0.33 × D_23_ | 3 | 5 | 3 | 0 | 1 | 12 |
|  | 4 | 6 | 0.67 × D_23_ | 5 | 0 | 2 | 2 | 2 | 11 |
|  | 5 | 6 | 0.33 × D_23_ | 4 | 0 | 2 | 2 | 2 | 10 |
|  | 5 | 7 | 0.67 × D_23_ | 2 | 2 | 1 | 0 | 1 | 6 |
|  | 6 | 7 | 0.33 × D_23_ | 5 | 7 | 1 | 1 | 2 | 16 |
|  | 6 | 8 | 0.33 × D_23_ | 5 | 1 | 2 | 0 | 2 | 10 |
| Chronic group | ≥7 | ≥8 | - | 126 | 41 | 80 | 22 | 62 | 331 |

A nonlinear course of spontaneous recovery was assumed during the early subacute phase of aphasia (1-3 months post onset) and a linear course of spontaneous recovery was assumed for the late subacute phase of aphasia (4-7 months post onset).

^a^Treatment cycle of 3 weeks only

GL = Global aphasia; WE = Wernicke aphasia; BR = Broca aphasia; AN = Anomic aphasia; NC = Not classified or nonstandard aphasia syndromes; syn = syndromes

**Table 3 Correlation coefficients between the three AAT assessments of the historical control group (n=96)**

|  | **Correlation between assessments at 1 and 4 months post onset** | | | **Correlation between assessments at 4 and 7 months post onset** | | |
| --- | --- | --- | --- | --- | --- | --- |
|  | **Pearson’s *r*** | **ICC (A,1)** | **ICC (C,1)** | **Pearson’s *r*** | **ICC (A,1)** | **ICC (C,1)** |
| Token Test | 0.851 | 0,757 | 0.851 | 0.949 | 0.929 | 0.946 |
| Repetition | 0.811 | 0.701 | 0.767 | 0.976 | 0.964 | 0.973 |
| Written Language | 0.854 | 0.735 | 0.853 | 0.957 | 0.942 | 0.955 |
| Naming | 0.862 | 0.736 | 0.853 | 0.943 | 0.925 | 0.938 |
| Comprehension | 0.784 | 0.641 | 0.771 | 0.934 | 0.913 | 0.931 |
| Profile level | 0.893 | 0.723 | 0.892 | 0.965 | 0.937 | 0.965 |

ICC (A,1) = Intraclass correlation coefficient Absolute Agreement

ICC (C,1) = Intraclass correlation coefficient Consistency

**Table 4 Raw score ranges and critical differences D_crit_ of the AAT subtests and profile level**

| **Subtest** | **Raw score range** | **Critical difference (one-tailed)^a^** | **Critical difference (two-tailed)^b^** |
| --- | --- | --- | --- |
| Token Test^c^ | 50-0 | 6.50 | 7.36 |
| Repetition | 0-150 | 13.61 | 15.42 |
| Written Language | 0-90 | 10.58 | 11.98 |
| Naming | 0-120 | 14.71 | 16.67 |
| Comprehension | 0-120 | 21.04 | 23.84 |
| Profile level^d^ | - | 1.10 | 1.41 |

Critical differences for significant improvement in a subtest were calculated one-tailed with *p* = 0.02 and two-tailed with *p* = 0.01 after Bonferroni correction for five subtest comparisons (type-I error level in psychometric single case analysis routinely = 0.10 to guard against unreasonably high type-II errors).

^a^D_crit_ = $z \times s_{x} \times\surd2(1-r_{tt})$ with *z* = 2.054 (98%-quantile of standard normal distribution for one-tailed test); *s_x_* = standard deviation of the respective subtest raw scores from the AAT validation study; *r_tt_*=reliability estimate (Cronbach alpha) of the respective subtest from the AAT validation study.

^b^D_crit_ = $z \times s_{x} \times\surd2(1-r_{tt})$ with *z* = 2.327 (99%-quantile of standard normal distribution for two-tailed test); *s_x_* = standard deviation of the respective subtest raw scores from the AAT validation study; *r_tt_*=reliability estimate (Cronbach alpha) of the respective subtest from the AAT validation study.

^c^Error score.

^d^*T* scores

**Table 5 Correlations of handedness and education with therapy responsiveness (responder/nonresponder)**

|  | ***n*** | **Cramer’s *V*** | ***p* value (χ^2^ test)** |
| --- | --- | --- | --- |
| Handedness | 286 | 0.038 | 0.811 |
| Education | 309 | 0.085 | 0.325 |

**Table 6 Comparison of pre- and post-treatment AAT subtests and profile level with raw scores after individual correction for spontaneous recovery and unadjusted raw scores with paired *t*-tests (one-tailed)**

|  | **Raw scores corrected for spontaneous recovery** | | | **Unadjusted raw scores** | | |
| --- | --- | --- | --- | --- | --- | --- |
|  | ***t* statistic** | ***p* value** | **Cohen’s *d***  **(95% CI)** | ***t* statistic** | ***p* value** | **Cohen’s *d***  **(95% CI)** |
| Token Test | -4.01 | <0.001* | 0.19 (0.10-0.28) | - 5.04 | <0.001* | 0.24 (0.14-0.33) |
| Repetition | -13.17 | <0.001* | 0.62 (0.52-0.72) | -14.08 | <0.001* | 0.67 (0.56-0.77) |
| Written Language | -10.79 | <0.001* | 0.51 (0.41-0.61) | -12.09 | <0.001* | 0.57 (0.47-0.67) |
| Naming | -12.99 | <0.001* | 0.61 (0.51-0.71) | -13.68 | <0.001* | 0.67 (0.54-0.75) |
| Comprehension | -8.02 | <0.001* | 0.38 (0.28-0.48) | - 9.10 | <0.001* | 0.43 (0.33-0.53) |
| Profile level | -15.04 | <0.001* | 0.71 (0.61-0.81) | -16.99 | <0.001* | 0.80 (0.70-0.80) |

CI = confidence interval

**p*<0.001

**Table 7 Results of one-sample tests against the weighted grand average (**$\boldsymbol{D)}$ **of the expected spontaneous recovery**

|  |  | **Early and late subacute groups (n=117)** | | | **Entire cohort (n=448)** | | |  |
| --- | --- | --- | --- | --- | --- | --- | --- | --- |
|  |  | $\boldsymbol{D}$ | ***p*-value** | **Effect size (95% CI)** | $\boldsymbol{D}$ | ***p*-value** | **Effect size (95% CI)** | |
| Token Test | *t*-test | 2.24 | 0.649 | -0.04 (-0.22-0.159) | 0.59 | 0.011* | 0.11 (0.02-0.20) | |
|  | Wilcoxon |  | 0.943 | -0.17 (-0.37-0.03) |  | 0.176 | 0.05 (-0.05-0.15) | |
| Repetition | *t*-test | 3.88 | <0.001** | 0.58 (0.38-0.77) | 1.01 | <0.001** | 0.57 (0.47-0.67) | |
|  | Wilcoxon |  | <0.001** | 0.60 (0.44-0.74) |  | <0.001** | 0.56 (0.47-0.64 | |
| Written Language | *t*-test | 4.10 | 0.023* | 0.19 (0.00-0.37) | 1.07 | <0.001** | 0.42 (0.32-0.51) | |
|  | Wilcoxon |  | 0.180 | 0.10 (-0.11-0.29) |  | <0.001** | 0.41 (0.31-0.50) | |
| Naming | *t*-test | 4.85 | <0.001** | 0.36 (0.17-0.55) | 1.27 | <0.001** | 0.52 (0.42-0.62) | |
|  | Wilcoxon |  | <0.001** | 0.37 (0.19-0.55) |  | <0.001** | 0.59 (0.46-0.63) | |
| Comprehen-sion | *t*-test | 3.49 | 0.135 | 0.10 (-0.08-0.28) | 0.91 | <0.001** | 0.33 (0.23-0.42) | |
|  | Wilcoxon |  | 0.133 | 0.12 (-0.1-0.32) |  | <0.001** | 0.39 (0.30-0.49) | |
| Profile level | *t*-test | 1.51 | <0.001** | 0.37 (0.18-0.56) | 0.40 | <0.001** | 0.58 (0.48-0.68) | |
|  | Wilcoxon |  | <0.001** | 0.39 (0.18-0.58) |  | <0.001** | 0.62 (0.54-0.70) | |

*P*-values based on parametric one sample *t*-tests and non-parametric one-sample Wilcoxon signed-rank tests against the respective $D$, one-tailed. Effect sizes with 95% confidence intervals are Cohen’s *d* for parametric tests and rank biserial correlation coefficient *r_c_* [30]. CI: confidence interval

*p<0.05

**p<0.001

**Table 8 Results of robust ANOVAs of the AAT subtests after correction for spontaneous recovery**

|  | **Interaction Time x Group** | | | **Main effect Time** | | | **Main effect Group** | | |
| --- | --- | --- | --- | --- | --- | --- | --- | --- | --- |
| **AAT subtest** | ***F (df)*** | ***p_uncorr_*** | ***p_corr_*** | ***F (df)*** | ***p_uncorr_*** | ***p_corr_*** | ***F (df)*** | ***p_uncorr_*** | ***p_corr_*** |
| Token Test | 1.08 (2,51.46) | 0.347 | 1.000 | 9.73 (1,61.9) | 0.003 | 0.003* | 4.69 (2,48.44) | 0.014 | 0.070 |
| Repetition | 0.59 (2,52.34) | 0.558 | 1.000 | 71.04 (1,71.79) | <0.001 | <0.001* | 1.44 (2,52.53) | 0.247 | 0.247 |
| Written Language | 0.22 (2,51.28) | 0.805 | 0.805 | 37.84 (1,54.60) | <0.001 | <0.001* | 3.32 (2,51.72) | 0.044 | 0.176 |
| Naming | 0.48 (2,48.59) | 0.624 | 1.000 | 64.25 (1,66.89) | <0.001 | <0.001* | 1.92 (2,52.54) | 0.157 | 0.314 |
| Comprehension | 0.51 (2,53.85) | 0.605 | 1.000 | 27.14 (1,70.62) | <0.001 | <0.001* | 2.2  (2,51.81) | 0.121 | 0.363 |

*p* values are reported uncorrected (uncorr) and Bonferroni-Holm corrected per effect (corr) for 5 ANOVAs.

**p* < 0.05, ***p* < 0.001 after Bonferroni-Holm correction

**Figure 1 Differential therapy effects between groups of chronicity for the Token Test.** Mean error scores from pre-treatment to post-treatment assessment in the early subacute, late subacute and chronic group. Error bars represent 95% CI for the variation in intraindividual changes [5].

**Figure 2 Differential therapy effects between groups of chronicity for the subtest Repetition.** Mean raw scores from pre-treatment to post-treatment assessment in the early subacute, late subacute and chronic group. Error bars represent 95% CI for the variation in intraindividual changes [5].

**Figure 3 Differential therapy effects between groups of chronicity for the subtest Written language.** Mean raw scores from pre-treatment to post-treatment assessment in the early subacute, late subacute and chronic group. Error bars represent 95% CI for the variation in intraindividual changes [5].

**Figure 4 Differential therapy effects between groups of chronicity for the subtest Naming.** Mean raw scores from pre-treatment to post-treatment assessment in the early subacute, late subacute and chronic group. Error bars represent 95% CI for the variation in intraindividual changes [5].

**Figure 5 Differential therapy effects between groups of chronicity for the subtest Comprehension.** Mean raw scores from pre-treatment to post-treatment assessment in the early subacute, late subacute and chronic group. Error bars represent 95% CI for the variation in intraindividual changes [5].

**References**

1. Huber W, Poeck K, Weniger D, Willmes K (1983) Aachener Aphasie Test. Hogrefe, Göttingen

2. Willmes K, Poeck K (1984) Ergebnisse einer multizentrischen Untersuchung über die Spontanprognose von Aphasien vaskulärer Ätiologie. Nervenarzt 55:62–71

3. Kertesz A, McCabe P (1977) Recovery patterns and prognosis in aphasia. Brain 100 Pt 1:1–18. https://doi.org/10.1093/brain/100.1.1

4. Mazzoni M, Vista M, Pardossi L, et al (1992) Spontaneous evolution of aphasia after ischaemic stroke. Aphasiology 6:387–396. https://doi.org/10.1080/02687039208248609

5. Cousineau D, Goulet M-A, Harding B (2021) Summary Plots With Adjusted Error Bars: The *superb* Framework With an Implementation in R. Advances in Methods and Practices in Psychological Science 4:1–18. https://doi.org/10.1177/25152459211035109
